# Prevalence and correlates of adolescent self-injurious thoughts and behaviors: A population-based study in Burkina Faso

**DOI:** 10.1177/00207640231175778

**Published:** 2023-06-16

**Authors:** Tracie I Ebalu, Jaclyn C Kearns, Lucienne Ouermi, Mamadou Bountogo, Ali Sié, Till Bärnighausen, Guy Harling

**Affiliations:** 1 Department of Psychology, University of Pittsburgh, PA, USA; 2 Department of Psychology, University of Rochester, NY, USA; 3 Centre de Recherche en Santé de Nouna, Burkina Faso; 4 Institute of Global Health, University Hospital, Heidelberg University, Germany; 5 Africa Health Research Institute, KwaZulu-Natal, South Africa; 6 Department of Global Health and Population, Harvard T.H. Chan School of Public Health, Boston, MA, USA; 7 Institute for Global Health, University College London, UK; 8 MRC/Wits Rural Public Health and Health Transitions Research Unit (Agincourt), University of the Witwatersrand, Johannesburg, South Africa; 9 School of Nursing and Public Health, College of Health Sciences, University of KwaZulu-Natal, Durban, South Africa

**Keywords:** Suicide, nonsuicidal self-injury, adolescence, sub-Saharan Africa

## Abstract

Self-injurious thoughts and behaviors (SITBs) are a growing concern among youth in sub-Saharan Africa, but their prevalence and correlates in this region are poorly understood. We therefore examined self-reported SITBs in a population-representative sample of youth in rural Burkina Faso. We used interviews from 1,538 adolescents aged 12 to 20 years living in 10 villages and 1 town in northwestern Burkina Faso. Adolescents were asked about their experiences with suicidal and nonsuicidal SITBs, adverse environmental factors, psychiatric symptoms, and interpersonal-social experiences. SITBs included lifetime prevalence of life is not worth living, passive suicide ideation, active suicide ideation, and nonsuicidal self-injury (NSSI). After describing SITB prevalence, we ran logistic and negative binomial regression models to predict SITBs. Weighted lifetime SITB prevalence estimates were: 15.6% (95% confidence interval [CI]: 13.7–18.0) for NSSI; 15.1% (95% CI: [13.2, 17.0]) for life is not worth living; 5.0% (95% CI [3.9, 6.0]) for passive suicide ideation; and 2.3% (95% CI [1.6, 3.0]) for active suicide ideation. Prevalence of life is not worth living increased with age. All four SITBs were significantly positively associated with mental health symptoms (depression symptoms, probable posttraumatic stress disorder) and interpersonal-social experiences (peer and social connectedness, physical assault, sexual assault and unwanted sexual experiences). Females were significantly more likely to report that their life was not worth living compared to males (aOR = 0.68; 95% CI [0.48, 0.96]). There is a high prevalence of SITBs among youth in rural Burkina Faso, most notably NSSI and life is not worth living, with interpersonal-social factors being the strongest predictors. Our results highlight the need for longitudinal SITB assessment to understand how risk for SITBs operates in resource-constrained settings, and to design interventions to mitigate risk. Given low school enrollment in rural Burkina Faso, it will be important to consider youth suicide prevention and mental health initiatives that are not school-based.

## Introduction

Self-injurious thoughts and behaviors (SITBs), a broad term that encompasses cognitions and actions related to both suicidal and nonsuicidal deliberate self-harm, are major public health concerns among youth globally. SITBs – most notably suicide – create significant costs to society (Florence et al., 2015), particularly in low- and middle-income countries (LMICs), which contain 90% of the world’s youth population and account for 75% of all suicide deaths (Saxena et al., 2014). To date, SITB research has almost exclusively focused on high-income countries with extremely limited coverage of countries in the sub-Saharan African (SSA) region. This paucity of research limits our understanding of the global burden of SITBs, including cross-national differences that may emerge in prevalence and risk correlates.

SITBs include thoughts such as passive suicide ideation (i.e., thoughts of wishing one were dead) and active suicide ideation (i.e., thoughts of killing oneself), as well as actions such as nonsuicidal self-injury (NSSI; i.e., hurting oneself on purpose with no intent to die) and a suicide attempt (i.e., intentional, non-fatal self-harm enacted with some intent to die) (Nock et al., 2008). According to the World Health Organization’s Global School-based Health Survey (GSHS), suicide ideation among youth in African countries was higher compared to other LMIC regions (McKinnon et al., 2016). Cross-national estimates found that select African countries (Saxena et al., 2014) had the highest prevalence of suicide death (20.4%) and suicide planning (i.e., selection of a specific method through one wishes to die; 23.7%), and the second highest prevalence of suicide attempt (19.3%) (Uddin et al., 2019) compared to countries in the Americas, Eastern Mediterranean, Europe, South-East Asia, and Western Pacific regions.

Despite expansions in the youth global mental health agenda (e.g., GSHS), research on SITB prevalence and risk correlates among SSA youth are still sparse. In terms of suicidal SITBs, the 12-month prevalence of suicide ideation ranges from 6% to 31% (Cluver et al., 2015; Glozah et al., 2018; Muula et al., 2007), and the prevalence of lifetime suicide attempt ranges from 7% to 26% (Cluver et al., 2015; Omigbodun et al., 2008; Oppong Asante & Meyer-Weitz, 2017). At present, there are no prevalence estimates of nonsuicidal SITBs among SSA youth. Research on risk correlates for suicidal SITBs among SSA youth is sparse and no research has yet to examine risk correlates for nonsuicidal SITBs. As a result of this limited research, this has left the field to draw upon risk correlates from other LMICs (as well as high-income countries), and then apply these to SSA youth without consideration of socioeconomic and cultural differentials.

Broadly, risk correlates for suicidal SITBs in LMICs include psychiatric symptoms (e.g., depressive symptoms), bullying, physical and sexual violence, loneliness, limited parental support, substance use, and child employment (Ibrahim et al., 2019; Kiss et al., 2015; McKinnon et al., 2016). Risk correlates for nonsuicidal SITBs in LMICs include low education/school absenteeism, lack of close friends, and physical abuse (Aggarwal et al., 2017). Current research on youth SITBs in SSA is unequally spread across the region, with several countries not covered, including Burkina Faso. Burkina Faso is a landlocked country in West Africa where almost half of the population lives under the poverty line (World Bank, n.d.; World Bank, 2017) and experiences food insecurity (Nanama & Frongillo, 2012). Burkina Faso also has one of the lowest Human Development index rankings, placing it at 182 out of 189 countries/territories, a metric that examines three dimensions of development (long and health life, access to knowledge, and a decent standard of living [*The next frontier*, United Nations Development Programme, 2020]). The population is primarily young with 70% under 25 years old (Ouedraogo, 2018), and nearly half work (42% of youth aged 5–14 work).

Currently, only a few studies on SITBs in Burkina Faso exist, focusing on specialized adult populations (sexual minority males and female sex workers) (Stahlman et al., 2016) and youth living in large towns (Nyundo et al., 2020). The lack of SITB research among youth living in rural and semi-rural communities in Burkina Faso is especially alarming given that approximately 71% of Burkinabé youth live in rural communities (Burkina Faso Rural Population, n.d.). Moreover, the lack of youth SITB research in Burkina Faso requires mental health providers to potentially misapply results from specialized adult populations to youth, a group experiencing significant and unique developmental changes during adolescence (Steinberg & Morris, 2001). SITBs have their onset in adolescence and we see the sharpest increase in suicide deaths between adolescence and young adulthood compared to any other age group (Nock et al., 2008), making this an important time for prevention and intervention. Taken together, SITB research on the prevalence and risk correlates of youth SITBs in Burkina Faso is crucial as it will provide insight into a key developmental period and inform effective prevention and intervention plans.

The present study aimed to address the aforementioned limitations by examining the prevalence and risk correlates of suicidal and nonsuicidal SITBs among Burkinabé youth. First, we examined the lifetime prevalence rate of suicidal (i.e., life is not worth living, passive suicide ideation, active suicide ideation, suicide plan, and suicide attempt) and nonsuicidal (i.e., NSSI) SITBs. Second, based on SITB risk correlates observed among youth in LMICs, we examined correlates such as demographic characteristics (e.g., gender), adverse environmental factors (e.g., food insecurity), interpersonal-social factors (e.g., lack of parental support), and mental health factors (e.g., depressive symptoms). Together, these results will extend our understanding of the epidemiology and risk for SITBs among Burkinabé youth.

## Methods

### Study setting and population

We used interview responses from a cohort of adolescents aged 12 to 20 years old living in rural and semi-rural Burkina Faso. This cohort is part of the Africa Research, Implementation Science and Education (ARISE) Adolescent Health Study, a collaboration of researchers across seven sub-Saharan African countries (Darling et al., 2020). The Burkina Faso study (ARISE Nouna) was conducted at the Nouna Health and Demographic Surveillance System (HDSS) site overseen by the Centre de Recherche en Santé de Nouna in Burkina Faso (Sié et al., 2010). The HDSS comprises the town of Nouna and 59 surrounding villages with a population of over 100,000 people.

The ARISE Nouna cohort was selected using a two-part stratified sampling method to ensure that the sample represented the HDSS in terms of ethnicity (and thus religion) and urbanicity. First, 10 Nouna HDSS villages were selected to ensure that all 5 main local ethnicities were included in the sample. Within these villages, a random sample of 1,795 youth aged 12 to 19 years old was drawn, based on a 2015 census of residents who were age-eligible on October 1, 2017. Second, a simple random sample of 749 age-eligible youth was drawn from one of the seven sectors of Nouna town. Interviews were conducted in November and December 2017 in the adolescents’ homes in French or other preferred local languages. Participants reported on sociodemographic characteristics, health and non-health behaviors, and health outcomes, using a questionnaire expanded from the GSHS (World Health Organization., n.d.). A random half of respondents used a nonverbal response card for sensitive questions, including SITB, NSSI, and violence-related questions (Harling et al., 2021). The nonverbal response card is a low-tech, low-literacy method that allows participants to provide non-verbal responses to sensitive questions without the interviewer learning what they said (Lindstrom et al., 2012).

### Ethics

Approvals for this study were obtained from the following: Institutional Ethics Committee of the Centre de Recherche en Santé de Nouna, village elders, participants (written consent/assent), and parent/guardian (written consent if participant >18 years old). ARISE Nouna was deemed exempt from institutional review by the Heidelberg Medical Faculty’s Ethics Committee due to the anonymized nature of all data received in Germany; University College London Research Ethics Committee approved secondary data analysis.

### Measures

**Figure 1.**
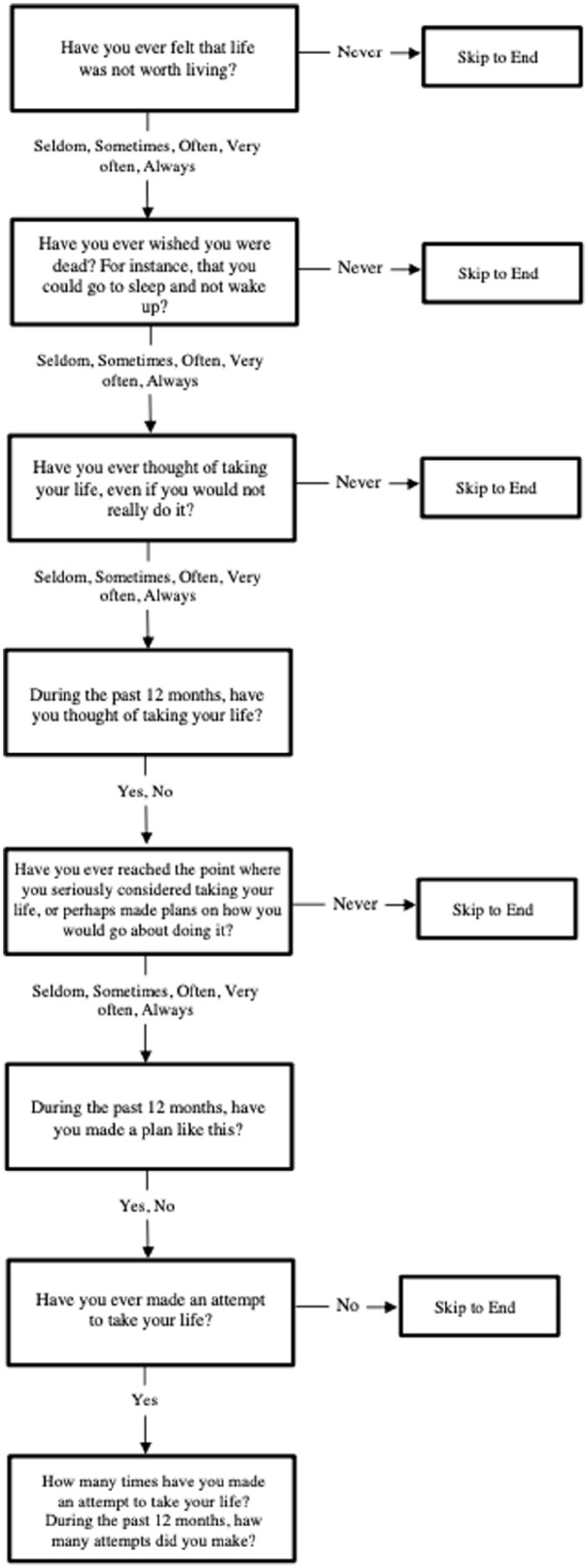
ARISE self-injurious thoughts and behaviors question series.

SITB questions were modeled after the 2003 GSHS questionnaire (World Health Organization, n.d.). Respondents were asked a series of questions, beginning with whether they thought their life was not worth living, then if they had ever wished they were dead (lifetime passive suicide ideation), if they had ever thought of taking their own life (lifetime active suicide ideation), as well as lifetime suicide planning and suicide attempt. Each question was asked only if the preceding response was affirmative (Figure 1). The NSSI questions were modeled after the Deliberate Self-Harm Inventory, which asks about intentional self-harm without the intent to die and frequency if ever done (Gratz, 2001).

For socio-demographics, we used binary sex and age in categories (12–13, 14–15, 16–17, 18+). For adverse environmental factors, we included household wealth in quintiles, past 12 months of employment, current school enrollment, highest educational attainment, and household food insecurity. For interpersonal-social factors, we included the number of close friends, peer belongingness (six levels), parental support (six levels), social connectedness (six levels), loneliness (six levels), as well as binary experiences of lifetime bullying victimization, physical assault, sexual assault, and unwanted sexual intercourse. Finally, for mental health factors, we included depression based on the six-item Kutcher Adolescent Depression Scale (KADS-6) (LeBlanc et al., 2002) and probable post-traumatic stress disorder (PTSD) based on the four-item Primary Care PTSD Screen for DSM-IV (PC-PTSD-IV) (Prins et al., 2004). See Supplemental Table 1 for variable details.

### Statistical analyses

Analyses were conducted using R (R Core Team, 2013) and Stata version 15.1 (Stata Corp, 2017). We excluded participants who were missing any outcome variables, after comparing excluded and non-excluded individuals. Descriptive statistics were calculated using median and interquartile ranges for continuous data and proportions for categorical data. Summary statistics were adjusted for survey non-response using inverse participation weights accounting for gender, ethnicity, and village/town. Differences between males and females for all variables were examined using Pearson chi-square for categorical variables and the Mann-Whitney *U* test for non-normally distributed continuous variables.

We then conducted logistic regression analyses in R to estimate the association between the predictor variables and any lifetime NSSI, life is not worth living, passive suicide ideation, and active suicide ideation. Since the 12-month NSSI frequency data were overdispersed, we ran a negative binomial regression analysis using Stata to evaluate the associations between the predictor variables and 12-month NSSI frequency. For each outcome, we included adverse environmental factors, interpersonal-social factors, and mental health factors in our models.

## Results

Of the 2,543 initial sample, 1,644 (64.6%) participants consented to participate in the study; 106 participants were removed from the analysis due to missing data (see Supplemental Table 2 for missingness comparison). We had a final sample of 1,538 participants (59.6% male, 40.4% female). There were significant differences between males and females in child employment (more male) and current school enrollment (more female). See Table 1 for sample characteristics.

**Table 1.** Descriptive characteristics of Burkinabé youth included in the study.

Categorical variables^a^	Total (*N* = 1,538)	Males (*n* = 916)	Females (*n* = 622)	*p*-Value
	Frequency (%)	Frequency (%)	Frequency (%)	
Age (years)				
12–13	473 (31%)	275 (30%)	198 (32%)	.919
14–15	399 (26%)	231 (25%)	168 (27%)	
16–17	342 (22%)	210 (23%)	132 (21%)	
18+	324 (21%)	200 (22%)	124 (20%)	
Household wealth index				
1 (least wealthy quintile)	297 (19%)	204 (22%)	93 (15%)	.419
2	300 (20%)	180 (20%)	120 (19%)	
3	335 (22%)	199 (22%)	136 (22%)	
4	287 (19%)	160 (17%)	127 (20%)	
5 (most wealthy quintile)	319 (21%)	173 (19%)	146 (23%)	
Food insecurity				
Never	1,377 (90%)	809 (88%)	568 (91%)	.899
Rarely	95 (6%)	65 (7%)	30 (5%)	
Sometimes	55 (4%)	35 (4%)	20 (3%)	
Most of the times	4 (<1%)	2 (<1%)	2 (<1%)	
Always	7 (<1%)	5 (1%)	2 (<1%)	
Child employment	959 (62%)	637 (70%)	322 (52%)	<.001
Currently in school	770 (50%)	426 (47%)	344 (55%)	.096
Ever been in school	1,148 (75%)	674 (74%)	474 (76%)	.593
Bullying	574 (37%)	369 (40%)	205 (33%)	.157
Physical assault	481 (31%)	292 (32%)	189 (30%)	.790
Sexual assault	51 (3%)	25 (3%)	26 (4%)	.490
Unwanted sexual experience	46 (3%)	26 (3%)	20 (3%)	.926
Loneliness				
Never	1,278 (83%)	779 (85%)	499 (80%)	.747
Rarely	114 (7%)	59 (6%)	55 (9%)	
Sometimes	110 (7%)	57 (6%)	53 (9%)	
Most of the time	22 (1%)	14 (2%)	8 (1%)	
Always	14 (1%)	7 (1%)	7 (1%)	
Depression	19 (1%)	11 (1%)	8 (1%)	1
PTSD	82 (5%)	35 (4%)	47 (8%)	.109
Continuous variables^b^	Median (IQR)	Median (IQR)	Median (IQR)	*p*-Value
Social network	3 (2, 5)	4 (2, 6)	2 (1, 4)	**.001**
School attainment	6 (0, 8)	6 (0, 8)	6 (2, 8)	.294
Parental support	2 (1, 2)	2 (1, 3)	1 (0, 2)	**<.001**
Social connectedness	4 (2, 4)	4 (3, 4)	4 (2, 4)	**<.001**

*Note.* PTSD = posttraumatic stress disorder.

a Group differences by sex were examined using Pearson chi-square tests.

b Group differences by sex were examined using Mann-Whitney *U* tests.

### Prevalence of SITBs

**Figure 2.**
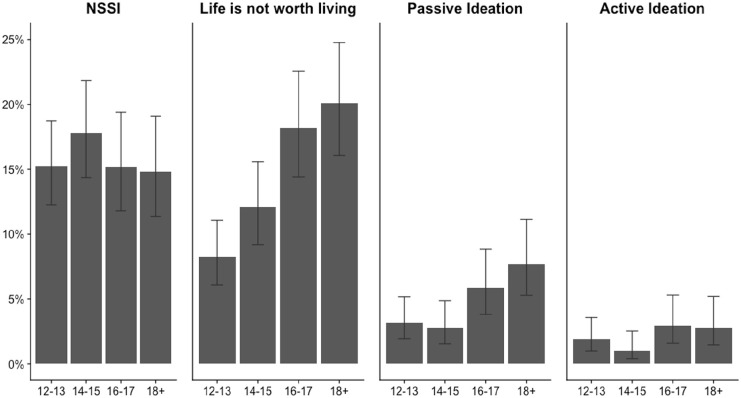
Lifetime prevalence of self-injurious thoughts and behaviors by age group. *Note.* Values are proportions with 95% confidence interval. NSSI = nonsuicidal self-injury.

Overall weighted SITB prevalences were: 15.6% (95% confidence interval [CI]: 13.7–18.0) for lifetime NSSI, 15.1% (95% CI [13.2, 17.0]) for lifetime life is not worth living, 5.0% (95% CI [3.9, 6.0]) for lifetime passive suicide ideation, 2.3% (95% CI [1.6, 3.0]) for lifetime active suicide ideation, and 2.0% (95% CI [1.4, 3.0]) for 12-month active suicide ideation (see Table 2). Prevalence of other SITBs was under 1%. Females were significantly more likely to report that their life was not worth living in their lifetime compared to males (19.0%, 95% CI [15.9, 23.0] vs. 11.4%, 95% CI [9.5, 14.0]). The prevalence of lifetime life is not worth living increased with age, but the prevalence of lifetime NSSI did not increase with age (Figure 2).

**Table 2.** Weighted prevalence rates of self-injurious thoughts and behaviors among 1,538 Burkinabe youth.

	Total (*N* = 1,538)	Males (*n* = 916)	Females (*n* = 622)	*p*-Value^a^
	Prevalence % (95% CI)	Prevalence % (95% CI)	Prevalence % (95% CI)	
Lifetime NSSI	15.6 [13.8, 18]	16 [13.7, 18]	15.1 [12.5, 18]	.714
Past 12 months NSSI frequency^b^				
0 times	24.9 [19.8, 31]	25.3 [19.1, 33]	24.6 [17.1, 34]	.211
1 time	27.2 [22.1, 33]	23.7 [17.5, 31]	30.8 [22.8, 40]	
2 times	21.1 [16.5, 27]	16.8 [11.7, 24]	25.6 [18.1, 35]	
3 times	9.8 [6.6, 14]	14 [9.3, 21]	5.5 [2.2, 13]	
⩾4 times	16.9 [12.7, 22)]	20.3 [14.8, 27]	13.5 [8.1, 22]	
Lifetime life is not worth living	15.1 [13.2, 17]	11.4 [9.5, 14]	19 [15.9, 23]	<.001
Lifetime passive SI	5.0 [3.9, 6]	4.6 [3.4, 6]	5.5 [3.8, 8]	.492
Lifetime active SI	2.3 [1.6, 3]	2.3 [1.5, 4]	2.3 [1.3, 4]	1
Past 12 months active SI	2.0 [1.4, 3]	1.9 (1.2–3)	2.1 [1.1, 4]	.973
Lifetime suicide plan	0.8 [0.4, 1]	0.7 [0.3, 1]	0.8 [0.3, 2]	1
Past 12 months suicide plan	0.6 [0.3, 1]	0.6 [0.3, 1]	0.7 [0.2, 2]	1
Lifetime suicide attempt	0.1 [0.02, 1]	0 (NA)	0.3 [0.04, 2]	NA
Past 12 months suicide attempt frequency	0.1 [0.02, 1]	0 (NA)	0.3 [0.04, 2]	NA

*Note*. NA is due to low prevalence, meaning that the value could not be calculated. CI = confidence interval; NSSI = nonsuicidal self-injury; SI = suicide ideation.

a Group differences were calculated using the Mann-Whitney *U* test for the continuous variables and chi-square analysis for the categorial variables.

b Frequency among those reporting any NSSI in the past 12 months.

### Variables associated with SITBs

In multivariable models, no environmental factor was significantly associated with lifetime NSSIs (Table 3), although those who had been in school but were not currently enrolled had almost a three-fold increased odds of past 12-month NSSI (adjusted Odds Ratio [aOR]: 2.70, 95% CI [1.51, 4.82]). Several interpersonal-social factors were significantly associated with lifetime and past 12-month NSSI after adjusting for all other factors. Larger social networks and more parental support were both associated with more NSSI, for example, for lifetime NSSI each additional close friend was associated with an 11% increase in odds (95% CI [1.07, 1.15]) and each additional level of parental support was associated with a 16% increased odds (95% CI [1.03, 1.31]). In contrast, greater social connectedness was associated with decreased past 12-month NSSI (aOR per additional level: 0.83, 95% CI [0.74, 0.93]). Past experience of physical or sexual violence were independently strongly predictive of both lifetime and past 12-month NSSI, with each more than doubling the adjusted odds of both outcomes. Among mental health factors, both depressive symptoms and probable PTSD were independently and significantly associated with lifetime and past 12-month NSSI after adjusting for all other factors. Each additional point on the KADS-6 scale was associated with 21% to 47% increased odds, and responses indicating probable PTSD associated with more than double the odds, of both NSSI outcomes.

**Table 3.** Multivariable regression analysis examining associations between predictor variables and suicidal injurious thoughts and behaviors among 1,538 Burkinabe youth.

Predictor variables^a^	Lifetime NSSI		12-month NSSI		Lifetime Life is not worth living		Lifetime passive SI		Lifetime active SI	
	aOR^b^	95% CI	aOR^b^	95% CI	aOR^b^	95% CI	aOR^b^	95% CI	aOR^b^	95% CI
Demographic characteristics										
Males (vs. females)	0.94	[0.68, 1.30]	0.93	[0.60, 1.50]	0.67	[0.47, 0.94]	0.98	[0.55, 1.76]	1.36	[0.56, 3.49]
Age group (vs. 12–13)										
14–15	1.23	[0.83, 1.82]	0.96	[0.58, 1.60]	1.48	[0.92, 2.39]	0.68	[0.29, 1.57]	0.34	[0.08, 1.21]
16–17	1.01	0.66, 1.55	1.13	0.64, 1.93	2.55	1.61, 4.07	1.49	[0.72, 3.16]	1.40	[0.52, 3.87]
18+	0.85	[0.53, 1.35]	0.81	[0.44, 1.49]	2.36	[1.46, 3.86]	1.21	[0.57, 2.62]	0.51	[0.15, 1.66]
Adverse environmental factors										
Household wealth index	0.95	[0.85, 1.06]	0.93	[0.81, 1.07]	1.01	[0.90, 1.13]	1.06	[0.87, 1.28]	0.94	[0.71, 1.25]
Child employment	0.93	[0.68, 1.28]	0.80	[0.54, 1.20]	0.88	[0.63, 1.23]	1.05	[0.60, 1.88]	0.66	[0.29, 1.54]
Currently in school	0.90	[0.62, 1.30]	0.67	[0.42, 1.07]	0.98	[0.67, 1.45]	0.48	[0.25, 0.91]	0.67	[0.27, 1.69]
Ever been in school	1.25	[0.81, 1.95]	2.70	[1.51, 4.82]	1.32	[0.85, 2.06]	1.18	[0.62, 2.26]	2.07	[0.72, 6.50]
Food insecurity	0.90	[0.67, 1.18]	0.79	[0.53, 1.17]	0.94	[0.70, 1.24]	0.96	[0.62, 1.41]	1.00	[0.57, 1.64]
Interpersonal-social factors										
Social network	1.11	[1.07, 1.15]	1.19	[1.11, 1.26]	0.99	[0.94, 1.03]	1.02	[0.95, 1.09]	1.05	[0.95, 1.15]
Parental support	1.16	[1.03, 1.31]	1.15	[0.98, 1.35]	1.05	[0.92, 1.19]	1.17	[0.95, 1.44]	1.23	[0.90, 1.69]
Social connectedness	0.83	[0.74, 0.93]	0.73	[0.62, 0.85]	0.87	[0.77, 0.99]	1.02	[0.82, 1.28]	1.13	[0.80, 1.69]
Loneliness	1.02	[0.82, 1.24]	0.79	[0.59, 1.06]	1.02	[0.83, 1.24]	1.25	[0.92, 1.65]	1.06	[0.61, 1.68]
Physical assault	2.79	[2.05, 3.80]	3.92	[2.55, 6.01]	0.85	[0.59, 1.21]	0.80	[0.42, 1.44]	0.73	[0.27, 1.79]
Sexual assault	1.46	[0.64, 3.14]	2.83	[1.10, 7.28]	1.64	[0.73, 3.51]	2.47	[0.89, 6.33]	2.97	[0.80, 10.0]
Unwanted sexual experiences	2.93	[1.31, 6.51]	2.81	[1.05, 7.50]	4.08	[1.88, 8.81]	6.36	[2.45, 15.9]	12.1	[3.52, 39.70]
Mental health factors										
Depressive symptoms	1.21	[1.09, 1.33]	1.47	[1.24, 1.75]	1.47	[1.33, 1.62]	1.27	[1.10, 1.45]	1.30	[1.05, 1.54]
PTSD cut-off	2.24	[1.26, 3.90]	2.22	[1.01, 4.90]	1.42	[0.77, 2.54]	1.92	[0.81, 4.25]	3.20	[1.00, 9.52]

*Note.* AOR = adjusted odds ratio; CI = confidence interval; NSSI = nonsuicidal self-injury; PTSD = posttraumatic stress disorder; SI = suicide ideation.

a All models are logistic regressions with the exception of the 12-month NSSI frequency model, which is a negative binomial regression.

b All analyses were adjusted for all other predictor variables.

Several factors were significantly associated with lifetime life is not worth living after adjusting for all other factors. These included gender, with males reporting this less often than females (aOR: 0.67, 95% CI [0.47, 0.94]) but no other environmental factors. For interpersonal social factors, each additional occurrence of an unwanted sexual experience was associated with a four-fold increased odds in lifetime life is not worth living (95% CI [1.88, 8.81]). Among mental health factors, depressive symptoms were significantly associated with lifetime life is not worth living; each additional point on the KADS-6 scale was associated with 47% increased odds of lifetime life is not worth living (95% CI [1.33, 1.62]).

Several interpersonal social factors were significantly associated with lifetime passive and active suicide ideation after adjusting for all other factors. For adverse environmental factors, current school enrollment was associated with significantly decreased lifetime passive ideation (aOR per additional level: 0.48, 95% CI [0.25, 0.91]) and non-significantly decreased active suicide ideation (aOR per additional level: 0.67, 95% CI [0.27, 1.69]) after adjusting for all other factors. Greater social connectedness (i.e., perceiving that others like having you around) was associated with a 13% increase in odds for lifetime active ideation (95% CI [0.80, 1.69]) per additional level. Both mental health factors were independently and significantly associated with suicide ideation after adjusting for all other factors. Each additional point on the KADS-6 scale was associated with 27% to 30% increased odds of each lifetime suicide ideation outcome. Probable PTSD was significantly associated with more than triple the odds for lifetime active suicide ideation.

## Discussion

We conducted a population-representative analysis of the prevalence and risk correlates of SITBs among youth living in rural and semi-rural Burkina Faso. Our study is one of the first among Burkinabé youth and one of the very few studies to examine SITBs in a highly resource-constrained setting. Lifetime prevalence of NSSI and feeling that life was not worth living were substantial (around 15% each), with the latter notably increasing with age – 20% of youth aged 18 to 20 endorsed that their life was not worth living compared to 8% of 12- to 13-year-olds. Approximately one in 20 and one in 40 Burkinabé youth endorsed passive and active suicide ideation in their lifetime respectively. A wide range of risk correlates, including interpersonal experiences, trauma exposure, and mental health symptoms – but few social and environment adversities – were significantly associated with SITBs.

The prevalence rates of NSSI and suicidal SITBs were comparable to those of other youth living in LMICs (Aggarwal et al., 2017; Uddin et al., 2019). In a recent review of LMICs, NSSI prevalence ranged from 11.5% to 35.8% (Mannekote Thippaiah et al., 2021), consistent with our findings, however, this review did not include any SSA countries, making it difficult to directly compare the NSSI estimates from SSA countries to our study sample. At present, there are no comparable prevalence rates of NSSI among SSA youth populations. The lifetime prevalence of passive and active ideation (5% and 2.3%) from our study was lower than estimates from the GSHS, which demonstrated an overall 12-month adolescent prevalence of 16.9% in LMICs, and 20.4% in Africa (Uddin et al., 2019). A direct comparison is, however, challenging since the GSHS asked all participants about suicide ideation, while our study only asked suicide ideation questions if respondents affirmed that they had ever felt that life was not worth living, potentially reducing reported prevalence. In contrast, the GSHS used past 12-month prevalence while we ask about lifetime prevalence, which should have increased reported values.

Our finding that females were more likely to report that their life is not worth living may reflect socio-cultural context (e.g., gender roles) – if girls have fewer life opportunities in rural Burkina Faso, this may increase the likelihood of feeling their lives are not worth living compared to boys (Brady et al., 2007; Crookston et al., 2021). Linked to this, the positive age gradient for reporting life is not worth living for both genders may reflect very limited social and economic opportunities in this highly resource-constrained setting, leading to adolescents feeling less hopeful about their futures as they move toward adulthood (Bigler et al., 2003; Wood et al., 2007).

We did not find significant gender differences in SITB prevalence except for life is not worth living. This lack of difference was unexpected, since past LMIC studies have found females more likely to report suicide ideation, suicide plan, and suicide attempt (Uddin et al., 2019). Again, our findings may reflect the filtered nature of our suicide ideation questions: if females were more likely than males to experience more serious SITBs without feeling life was not worth living, this would lead to underreporting of suicide ideation in females, explaining the difference between past work and our study.

Our findings that experiencing violence and mental health symptoms were associated with SITBs among Burkinabé youth were expected, given past evidence from adolescents in LMICs and high-income countries (Hadland et al., 2015; Wild et al., 2004). More surprisingly, adverse environmental factors such as household poverty and food insecurity did not significantly predict SITBs in our sample. This lack of association may reflect the widespread poverty in the study setting, such that these factors do not vary enough within the sample to predict SITBs.

Some of our findings regarding associations between interpersonal-social factors and SITBs were surprising. For example, SITBs were positively associated with both larger social networks (for NSSI) and social connectedness (for suicide ideation), despite social support typically being thought to protect against poor mental health outcomes including suicide ideation (Kleiman & Liu, 2013; Miller et al., 2015). Our finding may reflect peer effects, if larger social networks and social connectedness expose adolescents to others who express suicidal thoughts (Schlagbaum et al., 2021). Another surprising finding was that more parental support was associated with an increased risk for SITBs. This result contradicts past literature indicating that parental support predicts decreased likelihood of engaging in SITBs (Fong et al., 2022). We note that our parental support question asks about their attentiveness rather than direct supportive behavior, which may have had an unintended meaning in the social context. Certainly, all these results suggest the need for more research on social relationships and youth SITBs in heavily resource-constrained settings.

### Limitations

Our study has some notable strengths. For instance, we utilized a large sample of rural and semi-rural youth, a group that is rarely included in research on SITBs in SSA and LMIC. Further, our study is population representative, which allows us to better generalize to this setting. However, there are important limitations to this study. First, the SITB questionnaire structure may not have captured all adolescents who could have endorsed SITBs. Future studies would benefit from asking youth all questions to obtain the prevalence of the full spectrum of SITBs. Second, this study did not obtain information about age-of-onset for SITBs. Understanding the onset and temporal course of SITBs can provide valuable clinical information that can guide when prevention efforts may be most effective (Glenn et al., 2017). Third, this study is cross-sectional which limits our ability to determine causal direction between SITBs and their correlates. Future research can leverage longitudinal designs to understand if these factors drive SITBs (Kraemer et al., 1997).

## Conclusion

In this population-based study of 1,538 adolescents living in semi-rural and rural Burkina Faso, we found a substantive presence of SITBs, highlighting the importance of considering this health concern in Burkina Faso and other low-income settings. We also found a range of SITB correlates among Burkinabé youth, including interpersonal-social and mental health factors. These associations suggest potential avenues for interventions to treat and prevent SITBs, even in very low-income settings. Future research is needed in LMIC and SSA settings to longitudinally examine SITBs and their correlates (including capturing the age of onset), to understand the social-cultural influences on adolescent SITBs, and to develop more locally relevant interventions. Such work has the potential to address current harmful behavior and prevent future increases.

## Supplemental Material

sj-docx-1-isp-10.1177_00207640231175778 – Supplemental material for Prevalence and correlates of adolescent self-injurious thoughts and behaviors: A population-based study in Burkina Faso Click here for additional data file. Supplemental material, sj-docx-1-isp-10.1177_00207640231175778 for Prevalence and correlates of adolescent self-injurious thoughts and behaviors: A population-based study in Burkina Faso by Tracie I Ebalu, Jaclyn C Kearns, Lucienne Ouermi, Mamadou Bountogo, Ali Sié, Till Bärnighausen and Guy Harling in International Journal of Social Psychiatry
