# Supplementary material for: Prevalence and correlates of adolescent self-injurious thoughts and behaviors: A population-based study in Burkina Faso
Source: Int J Soc Psychiatry. 2023 Jun 16;69(7):1626–35. doi: 10.1177/00207640231175778 (PMC10657509; doi:10.1177/00207640231175778)
Supplement: sj-docx-1-isp-10.1177_00207640231175778 – Supplemental material for Prevalence and correlates of adolescent self-injurious thoughts and behaviors: A population-based study in Burkina Faso [file sj-docx-1-isp-10.1177_00207640231175778.docx]

**Prevalence and correlates of adolescent self-injurious thoughts and behaviors: A population-based study in Burkina Faso**

**Supplemental Table 1.**

Study variables

| **Variable** | **Interview Question/Measure (Response Options)** | **Modifications made for analyses** |
| --- | --- | --- |
| ***Demographic Characteristics*** | | |
| Sex (Gender) | What is your sex? *(Male, Female)* | None |
| Age | How old are you? *(Free response, in years)* | Four age group categories were created: 12-13, 14-15, 16-17, 18+ |
| ***Self-Injurious Thoughts and Behaviors (SITBs)^1^*** | | |
| Life not worth living | Have you ever felt that life was not worth living? *(Never, Seldom, Sometimes, Often, Very Often, Always)* | Likert rating codes as integer |
| Passive suicide ideation | Have you ever wished you were dead? For instance, that you could go to sleep and not wake up? *(Never, Seldom, Sometimes, Often, Very Often, Always)* | Likert ratings codes as integer |
| Active suicide ideation | Lifetime: Have you ever thought of taking your life, even if you would not really do it? *(Never, Seldom, Sometimes, Often, Very Often, Always)* | Integer |
|  | Past 12 months: During the past 12 months, have you thought of taking your own life? (*Yes, No*) | Binary (yes, no) |
| Suicide plan | Lifetime: Have you ever reached the point where you seriously considered taking your life, or perhaps made plans on how you would go about doing it? (*Never, Seldom, Sometimes, Often, Very Often, Always)* | Integer |
|  | Past 12 months: During the past 12 months, have you made a plan like this? *(Yes, No)* | Binary (yes, no) |
| Suicide attempt | Lifetime: Have you ever made an attempt to take your life? (*Yes, No*) | Binary (yes, no) |
|  | Lifetime Frequency: How many times have you made an attempt to take your life? *(Free response; integer)* | Integer |
|  | Past 12 months: During the past 12 months, how many attempts did you make? *(Free response; integer)* | Integer |
| ***Adverse Environmental Factors*** | | |
| Household wealth | Household wealth was calculated from the leading component of a principal component analysis of household assets | 1 (least wealthy quintile) to 5 (most wealthy quintile). |
| Child employment | Have you done any work in the last 12 months? By work, we mean any activity to earn money or obtain food? (*Yes, No*) | Binary (yes, no). |
| School enrollment | Are you currently in school? (*Yes, No*) | Binary (yes, no) |
| Overall school attainment | What's the highest level of school you attended? *(None, Primary [1-6],Post-Primary [7-10], Secondary [1-3], Technical/Vocational, University, Don't Know)* | Likert rating codes as integer |
| Food insecurity | During the past 30 days, how often did you go hungry because there was not enough food in your home? (*Never, Seldom, Sometimes, Often, Very Often, Always)* | Likert rating codes as integer |
| ***Interpersonal-Social Factors*** | | |
| Social network | How many close friends do you have? *(Free response; integer)* | Likert rating codes as integer |
| Parental support | How often do your parents/guardians pay attention to your opinion or what you say? (*Never, Seldom, Sometimes, Often, Very Often, Always)* | Likert rating codes as integer |
|  | How often do your parents/guardians take time to talk with you about things that happened to you? (*Never, Seldom, Sometimes, Often, Very Often, Always)* | Likert rating codes as integer |
| Social connectedness | How much of the time would you say people your age like having you in the group? (*Never, Seldom, Sometimes, Often, Very Often, Always)* | Likert rating codes as integer |
| Loneliness | During the past 12 months, how often have you felt lonely? (*Never, Seldom, Sometimes, Often, Very Often, Always)* | Likert rating codes as integer |
| Bullying | Have you ever been bullied? (*Yes, No*) | Presence/absence was coded as binary |
| Experiences of violence | In all your life, have you experienced any of the following events? physical assault, sexual assault, or other unwanted sexual experience. | Presence/absence was coded as binary |
| ***Mental Health Factors*** | | |
| Depression | Depression symptoms in the past week were assessed using the 6-item Kutcher Adolescent Depression Scale (KADS) | Sum of all 6 items KADs scale |
| Posttraumatic stress disorder (PTSD) | PTSD symptoms in the past 30 days were assessed with the four-item Primary Care PTSD Screen for DSM-IV (PC-PTSD-IV) | Probable PTSD was defined as a score of ≥ 3 on the PC-PTSD-IV |

^1^ For SITBs, respondents were asked a series of questions, beginning with whether they had thought life was not worth living, then if they had ever wished they were dead (lifetime passive suicide

ideation), if they had ever thought of taking their own life (lifetime active suicide ideation), as well as lifetime suicide planning and suicide attempt. Each question was posed only if the preceding response was affirmative. NSSI questions were modeled after the Deliberate Self-Harm Inventory (Gratz, 2001b) asking two questions, one on lifetime intentional self-injury and the other on frequency of self-harm in the past 12 months.

**Supplemental Table 2.**

Comparison of non-missing and missing descriptive characteristics included in the study

|  | Total  (*N* = 1644) | | Without Missing  (*n* = 1538) | | Missing  (*n* = 106) | *p*-value |  |  |
| --- | --- | --- | --- | --- | --- | --- | --- | --- |
|  | Frequency (%) | | Frequency (%) | | Frequency (%) |  |  |  |
| ***Categorical Variables^1^*** | |  | | | | | |  |
| **Age (years)** | |  | | | | | |  |
| 12-13 | | 520 (32%) | | 473 (31%) | | 47 (44%) | 0.047 |  |
| 14-15 | | 424 (26%) | | 399 (26%) | | 25 (24%) |  |  |
| 16-17 | | 361 (22%) | | 342 (22%) | | 19 (18%) |  |  |
| 18+ | | 339 (21%) | | 324 (21%) | | 15 (14%) |  |  |
| **Household Wealth Index** | |  | |  | |  |  |  |
| 1 (least wealthy quintile) | | 331 (20%) | | 297 (19%) | | 34 (32%) | <0.001 |  |
| 2 | | 331 (20%) | | 300 (20%) | | 31 (29 %) |  |  |
| 3 | | 355 (22%) | | 335 (22%) | | 20 (19%) |  |  |
| 4 | | 299 (18%) | | 287 (19%) | | 12 (11%) |  |  |
| 5 (most wealthy quintile) | | 328 (20%) | | 319 (21%) | | 9 (8%) |  |  |
| **Food Insecurity** | |  | |  | |  |  |  |
| Never | | 1454 (88%) | | 1377 (90%) | | 77 (73%) | <0.001 |  |
| Rarely | | 112 (7%) | | 95 (6%) | | 17 (16%) |  |  |
| Sometimes | | 63 (4%) | | 55 (4%) | | 8 (8%) |  |  |
| Most of the time | | 7 (<1%) | | 4 (<1%) | | 3 (3%) |  |  |
| Always | | 7 (<1%) | | 7 (<1%) | | 0 (0%) |  |  |
| **Child Employment** | | 1008 (61%) | | 49 (62%) | | 49 (46%) | 0.125 |  |
| **Currently in School** | | 822 (50%) | | 770 (50%) | | 52 (49%) | 0.930 |  |
| **Ever been in School** | | 1226 (75%) | | 1148 (75%) | | 78 (74%) | 0.911 |  |
| **Bullying** | | 608 (37%) | | 574 (37%) | | 34 (32%) | 0.548 |  |
| **Physical Assault** | | 507 (31%) | | 481 (31%) | | 26 (25%) | 0.647 |  |
| **Sexual Assault** | | 60 (4%) | | 51 (3%) | | 9 (8%) | 0.004 |  |
| **Unwanted Sex** | | 59 (4%) | | 46 (3%) | | 13 (12%) | <0.001 |  |
| **Loneliness** | |  | |  | |  |  |  |
| Never | | 1357 (83%) | | 1278 (83%) | | 79 (75%) | 0.637 |  |
| Rarely | | 126 (8%) | | 114 (7%) | | 12 (11%) |  |  |
| Sometimes | | 118 (7%) | | 110 (7%) | | 8 (8%) |  |  |
| Most of the time | | 23 (1%) | | 22 (1%) | | 1 (<1%) |  |  |
| Always | | 15 (1%) | | 14 (1%) | | 1 (<1%) |  |  |
| **Depression** | | 20 (1%) | | 19 (1%) | | 1 (<1%) | 1 |  |
| **PTSD** | | 89 (5%) | | 82 (5%) | | 7 (7%) | 0.422 |  |
| ***Continuous Variables*^2^** | | | | | | | |  |
|  | | | Median (IQR) | Median (IQR) | Median (IQR) | | *p*-value | |
| **Social Network** | | | 3 (2, 5) | 3 (2, 5) | 4 (1.8, 6) | | 0.699 | |
| **School Attainment** | | | 6 (0, 8) | 6 (0, 8) | 5 (0, 6) | | 0.213 | |
| **Parental Support** | | | 2 (1, 2) | 2 (1, 2) | 1 (0, 2) | | 0.029 | |
| **Social Connectedness** | | | 4 (2, 4) | 4 (2, 4) | 4 (2, 4) | | 0.768 | |

^1^Group differences by sex were examined using Pearson chi-square tests.

^2^Group differences by sex were examined using Mann Whitney U tests.

*Note.* PTSD = posttraumatic stress disorder.
